# Classification and transfer learning of sleep spindles based on convolutional neural networks

**DOI:** 10.3389/fnins.2024.1396917

**Published:** 2024-04-24

**Authors:** Jun Liang, Abdelkader Nasreddine Belkacem, Yanxin Song, Jiaxin Wang, Zhiguo Ai, Xuanqi Wang, Jun Guo, Lingfeng Fan, Changming Wang, Bowen Ji, Zengguang Wang

**Affiliations:** ^1^Department of Rehabilitation Medicine, Tianjin Medical University General Hospital, Tianjin, China; ^2^Department of Computer and Network Engineering, College of Information Technology, UAE University, Al Ain, United Arab Emirates; ^3^School of Nursing, Tianjin Medical University, Tianjin, China; ^4^School of Medicine, Tianshi College, Tianjin, China; ^5^Zouping Traditional Chinese Medicine Hospital, Zouping, China; ^6^People’s Hospital of Xianghe Country, Langfang, China; ^7^Unmanned System Research Institute, Northwestern Polytechnical University, Xi’an, China; ^8^National Key Laboratory of Unmanned Aerial Vehicle Technology, Northwestern Polytechnical University Xi’an, Xi’an, China; ^9^Key Laboratory of Complex System Control Theory and Application, Tianjin University of Technology, Tianjin, China; ^10^Xuanwu Hospital, Capital Medical University, Beijing, China

**Keywords:** sleep spindles, electroencephalogram, convolutional neural network, polysomnography, transfer learning

## Abstract

**Background:**

Sleep plays a critical role in human physiological and psychological health, and electroencephalography (EEG), an effective sleep-monitoring method, is of great importance in revealing sleep characteristics and aiding the diagnosis of sleep disorders. Sleep spindles, which are a typical phenomenon in EEG, hold importance in sleep science.

**Methods:**

This paper proposes a novel convolutional neural network (CNN) model to classify sleep spindles. Transfer learning is employed to apply the model trained on the sleep spindles of healthy subjects to those of subjects with insomnia for classification. To analyze the effect of transfer learning, we discuss the classification results of both partially and fully transferred convolutional layers.

**Results:**

The classification accuracy for the healthy and insomnia subjects’ spindles were 93.68% and 92.77%, respectively. During transfer learning, when transferring all convolutional layers, the classification accuracy for the insomnia subjects’ spindles was 91.41% and transferring only the first four convolutional layers achieved a classification result of 92.80%. The experimental results demonstrate that the proposed CNN model can effectively classify sleep spindles. Furthermore, the features learned from the data of the normal subjects can be effectively applied to the data for subjects with insomnia, yielding desirable outcomes.

**Discussion:**

These outcomes underscore the efficacy of both the collected dataset and the proposed CNN model. The proposed model exhibits potential as a rapid and effective means to diagnose and treat sleep disorders, thereby improving the speed and quality of patient care.

## 1 Introduction

Sleep plays a critical role in an individual’s life and work as it occupies one-third of the 24 h in a day. Adequate sleep is essential to maintain a refreshed state of mind and optimal performance in personal and professional aspects. In the short term, insufficient sleep can lead to impaired memory and attention, and over longer periods, it can induce a range of physical and mental health conditions, e.g., hypertension, diabetes, and Alzheimer’s disease. In severe cases, insufficient sleep can result in neurological disorders or even death. Previous studies have demonstrated that sleep disorders are showing an increasing trend and are expected to continuously grow (Stranges et al., 2012).

Sleep spindles manifest as bursts of narrow-band activity that prominently feature in the electroencephalography (EEG) signals acquired across one or multiple scalp regions. Ranging from 11–16 Hz in frequency, they serve as a significant marker of nonrapid eye movement stage 2 sleep. Sleep spindles are an important marker for humans to enter sleep (Iber et al., 2007; LaRocco et al., 2018), and they play a crucial role in protecting the sleeping brain from external sensory stimuli and serve as a biomarker of sleep integrity (Dang-Vu et al., 2010; Saletin and Walker, 2013; Thanh et al., 2015). Studies have identified a close relationship between sleep spindles and other sleep-related EEG rhythms, as well as between spindles and synaptic plasticity (Ulrich, 2016). Increasing evidence suggest that neurologic and psychiatric disorders, e.g., Parkinson’s disease (Christensen et al., 2014; Latreille et al., 2015) and schizophrenia (Wamsley et al., 2012), are associated with decreased memory function and reduced spindle activity during sleep (Ferrarelli et al., 2010; Latreille et al., 2015). Similarly, the decline in learning ability in the elderly is related to decreased spindle activity in the prefrontal cortical region (Lu and Göder, 2012). Thus, by enhancing the study of sleep spindles, a deeper understanding of human sleep patterns and physical diseases can be acquired, which is expected to facilitate timely medical diagnoses and treatments.

Deep learning methods are widely used to identify and classify sleep spindles. For example, in 2017, Athanasios and Clifford (2015) proposed an automatic detection method for sleep spindles based on a wavelet algorithm, which was verified on the MASS (Montreal Archive of Sleep Studies) and DREAMS (Dreams Sleep Spindle Database) datasets. This method achieved sensitivity of 84 and 76% and specificity of 90 and 92% on the MASS and DREAMS datasets, respectively. In addition, Lachner-Piza et al. (2018) proposed a new single-channel spindle classification method that achieved a sensitivity of 53% and a precision of 37% on the DREAMS dataset. This method also achieved sensitivity and precision values of 77% and of 46% on the MASS dataset, respectively. In 2019, Kulkarni et al. (2019) proposed SpindleNet, which achieved the highest F1 score of 0.75 and AUC (area under curve) values of 0.9897 on different sleep datasets on different species with different ages; however, only a single channel was used in this study. In 2020, Kinoshita et al. (2020) employed the synchronous squeezed wavelet transform method to detect spindles combined with the random under-sampling boosting method and achieved a sensitivity of 76.9%. In 2021, Chen et al. (2021) proposed an automatic spindle detection algorithm combining EEG features, which achieved an F1 score of 0.66. In addition, Chen et al. (2023) proposed a fusion algorithm for spindle wave detection that achieved accuracy and precision of 90.4 and 91.6%, respectively.

Transfer learning involves transferring parameters of a pretrained model to design and train a completely new network, which can conserve computational resources and reduce training time (Öztürk et al., 2023). The transfer network structure is divided into two parts, i.e., the pretrained network and the transfer network. In this study, a pretrained network learned rich feature representations of spindle data, and the extracted features achieved excellent classification performance. The parameters in the network only need to be fine-tuned to adapt to new spindle data. Thus, in the case of insufficient EEG samples, transfer learning is a very convenient and effective method for training deep neural networks (Öztürk et al., 2023), which can increase model convergence speed, enhance model generalizability, and improve model performance on new tasks (Pan and Yang, 2010).

The characteristics of sleep spindles, e.g., power, duration, and frequency, vary with age, health conditions, and species (Purcell et al., 2017); thus, annotating spindles from EEG data across populations is costly. Considerable variations in the amplitude, duration, and frequency statistics of EEG or spindle data may exist among different EEG recordings. Therefore, this study examines the feasibility of employing transfer learning techniques in the study of sleep spindles, aiming to discern the efficacy of transferring knowledge gleaned from the spindle data of healthy subjects to individuals with insomnia. To the best of our knowledge, few studies have investigated the use of transfer learning in sleep spindle research. Kulkarni et al. (2019) conducted preliminary research on the application of transfer learning in studying sleep spindles and demonstrated the robustness of transfer learning methods across different subjects, age and health groups, and species.

This study proposes an innovative method to analyze spindles, which can be applied to different types and sources of datasets, yielding new possibilities for sleep research and clinical applications. The proposed method is particularly effective at handling unlabeled data and can provide valuable insights for advances in the field of sleep neuroscience. In addition, our findings are expected to provide support for broader sleep research and medical diagnosis.

This study focuses on the application of transfer learning in sleep spindle research. We collected sleep data from 20 healthy subjects and 10 insomnia subjects and annotated the spindles in the C3 and C4 channels. A new convolutional neural network (CNN) model is proposed to classify the data from the healthy and insomnia subjects, achieving satisfactory results. Then, through transfer learning, the model trained on the data from healthy subjects was applied to the data from the insomnia subjects. Two methods, i.e., partial and full transfer of the convolutional layers, were explored during the transfer process, and their classification results were compared. The findings demonstrate that partial transfer of the convolutional layers realized better adaptability to the new task while maintaining high classification accuracy. The findings of this study provide an effective approach to improve sleep spindle detection performance using transfer learning.

## 2 Data collection and feature analysis

### 2.1 Data collection and preprocessing

In this study, data collection took place at the Tianjin Medical University General Hospital and the Xuanwu Hospital of Capital Medical University. A cohort of 20 healthy participants (nine males and eleven females; average age of 40.2 years) and 10 participants with insomnia (six males and four females; average age of 38.6 years) were recruited for the EEG data acquisition process. Prior to conducting the experiment, the subjects were surveyed to acquire basic information, including gender, age, insomnia status, and insomnia duration. In addition, all participants were evaluated using the Pittsburgh Sleep Quality Index (PSQI) questionnaire. Table 1 shows the specific information of the 30 subjects. Note that a higher PSQI score indicates poorer sleep quality, where a score of 11 or higher indicates the insomnia, and lower scores are considered to be normal.

**Table 1:** Detailed information of the normal and insomnia subjects.

Healthy	Sex	PSQI	Age	Healthy	Sex	PSQI	Age	Insomnia	Sex	PSQI	Age
1	Male	4	27	11	Female	4	36	1	Male	17	39
2	Female	2	27	12	Male	1	59	2	Female	20	42
3	Female	3	27	13	Female	3	35	3	Male	17	27
4	Female	2	47	14	Male	1	41	4	Female	20	24
5	Female	3	40	15	Male	2	32	5	Female	17	26
6	Female	2	39	16	Male	2	47	6	Female	20	60
7	Female	2	52	17	Male	1	26	7	Female	17	54
8	Female	1	26	18	Female	2	31	8	Male	20	40
9	Male	2	46	19	Female	3	46	9	Female	17	52
10	Female	2	60	20	Male	2	60	10	Male	17	46

**Figure 1:**
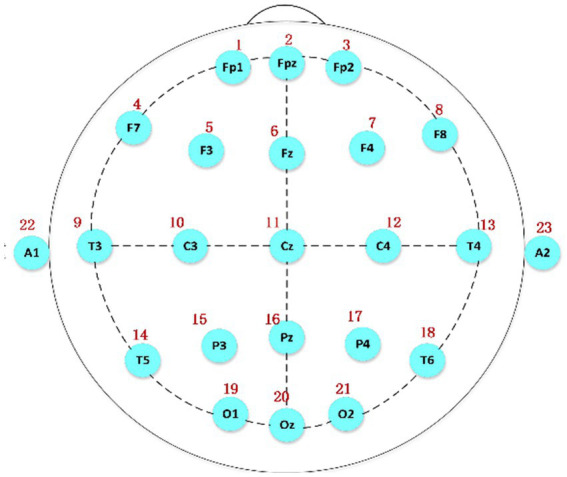
The electrode placement system based on the international 10/20 standard, A1 and A2 serving as reference electrodes.

Polysomnography was employed to collect the relevant data. Figure 1 shows the international 10/20 standard electrode placement system (Herwig et al., 2003), which was used to determine the electrode positions of the relevant EEG signals collected in this paper.

The data used in this study were acquired from the C3 and C4 channels because spindles appear most frequently in these two central brain regions. In addition, these two channels are relatively close to each other, and the results are similar, which can be used as a reference and for further verification of the accuracy of the experimental data.

The EEG technicians annotated spindles in the experimental data, along with the channels where the spindles were located, start time of spindle, and duration of spindle. Among the used channels, the C3 and C4 channels contained a higher number of spindles, which were relatively easy to identify. Based on the start time and duration marked by the technicians, we found the midpoint of each spindle and extended it by 1.5 s to the left and right to obtain a 3 s data segment. Then, we obtained negative samples that did not contain spindles based on the part of the entire night’s sleep data without annotations. Note that variations in the testing room could lead to differences in the number of spindles due to differences in the external environment. Thus, when obtaining negative samples, it was necessary to obtain continuous 3 s segments equal to the number of annotated positive samples from the same channel of the same subject. After the above operations, we obtained a balanced set of positive and negative samples for subsequent experimentation. The data acquisition process was the same for the insomnia subjects. A total of 31,718 spindles were obtained in the dataset of the normal subjects. In the dataset of the insomnia subjects, there were 13,960 spindles.

The collected data were preprocessed by MNE (Andersen, 2018). In the experiment, the sampling frequency was 1,024 Hz. For convenience, the data were downsampled to 100 Hz. Then, the FIR (Finite Impulse Respond Filter) (Gupta and Kumar, 2021) was applied to the collected EEG data using a Hamming window as a sliding window with a passband range of 0.3–30 Hz. Finally, the obtained data was *z*-score normalized.

### 2.2 Feature analysis

We conducted a detailed comparative analysis of the spindle and nonspindle data in both the time and frequency domains between the healthy subjects and insomnia subjects.

**Figure 2:**
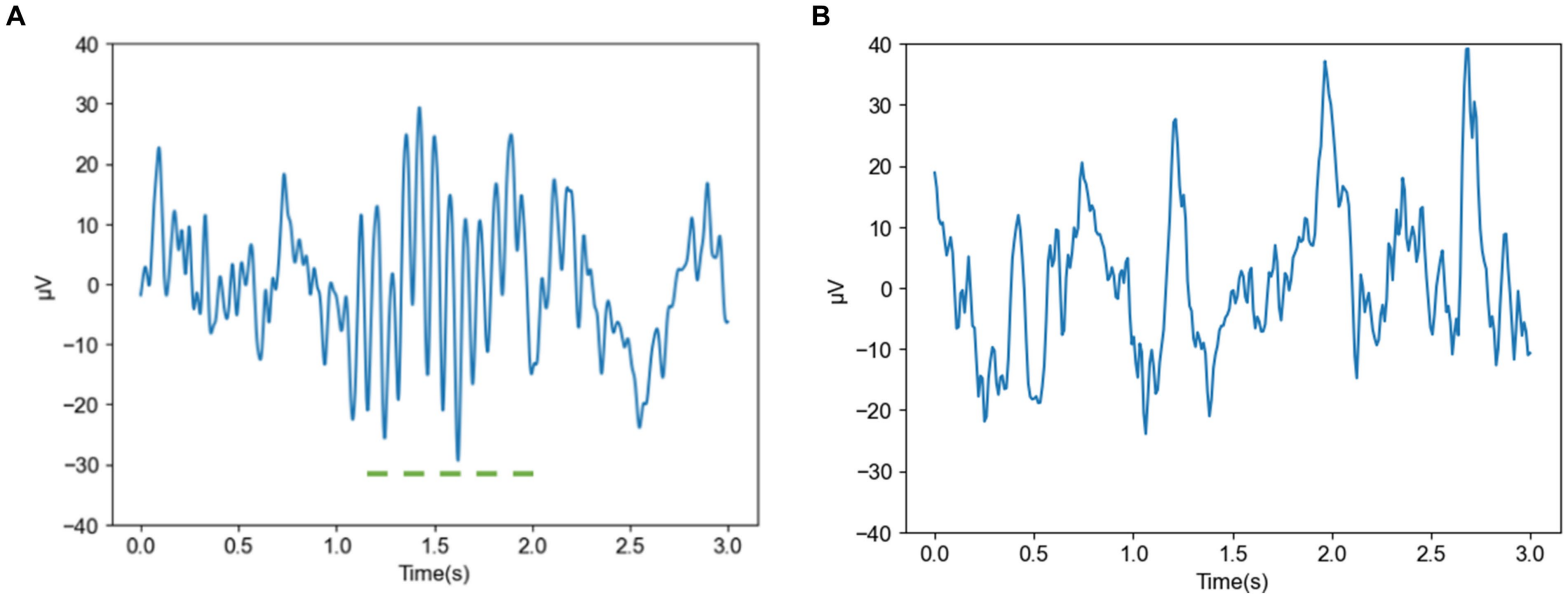
Spindle and nonspindle data of a normal subject **(A)** Spindle of a normal subject **(B)** Nonspindle of a normal subject.

Figures 2A,B show the spindle and nonspindle data of a normal subject, respectively. As shown, the amplitude of the spindle ranges from −30 to 30 μV, with a distinct spindle segment lasting approximately 1 s. In addition, the amplitude fluctuation of the nonspindle data differs from the spindle data, and the shape of the nonspindle data differs significantly from the spindle data.

**Figure 3:**
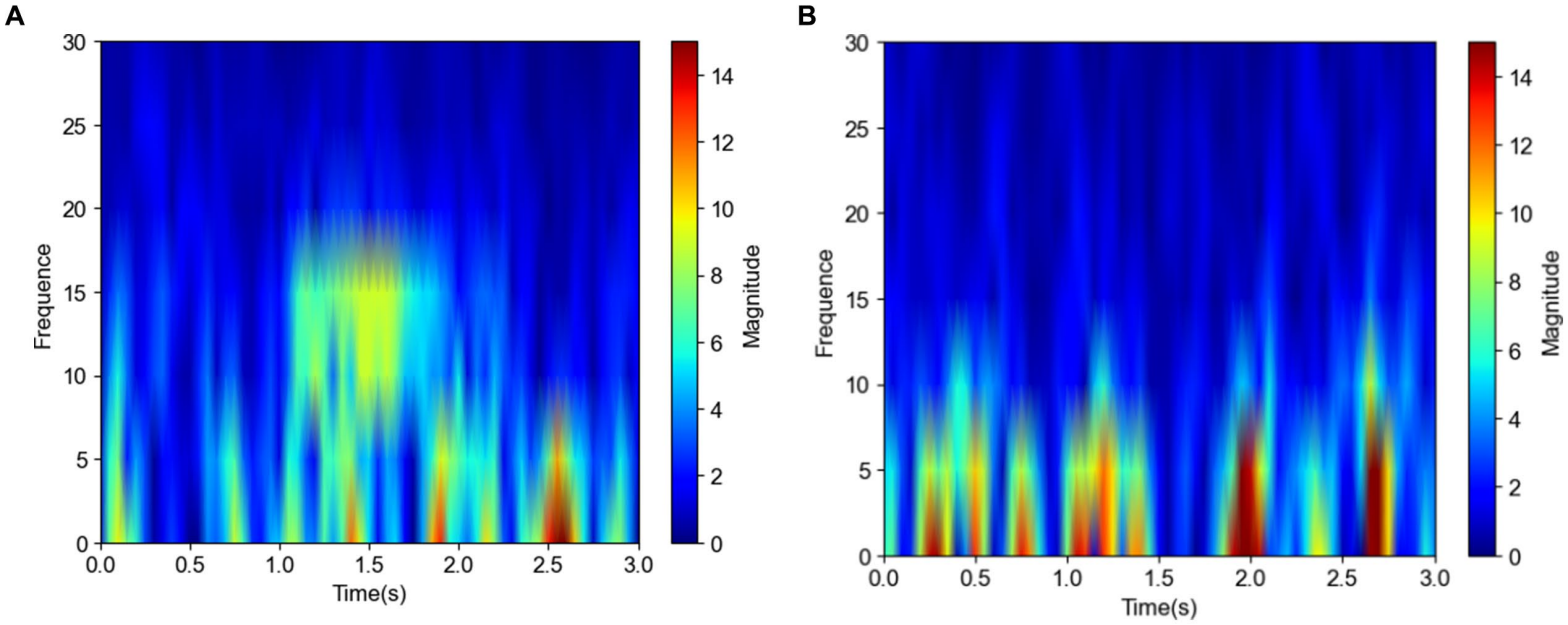
Frequency domain plots of the spindle and nonspindle in a normal subject **(A)** Frequency domain of a spindle **(B)** Frequency domain of a nonspindle.

Figures 3A,B show the frequency domain plots of a spindle and a nonspindle in a normal subject, respectively, where colors closer to red indicate higher energy. Compared with the nonspindle, the spindle is clearer and more distinguishable. With increasing energy, the color transitions from blue to red, which indicates a significant increase in energy concentration in the sigma band of 10–18 Hz. The frequency domain of the nonspindle (Figure 3B) shows no obvious increase in energy above 10 Hz compared to the frequency domain of the spindle, and there is a red display of energy between 0–10 Hz.

**Figure 4:**
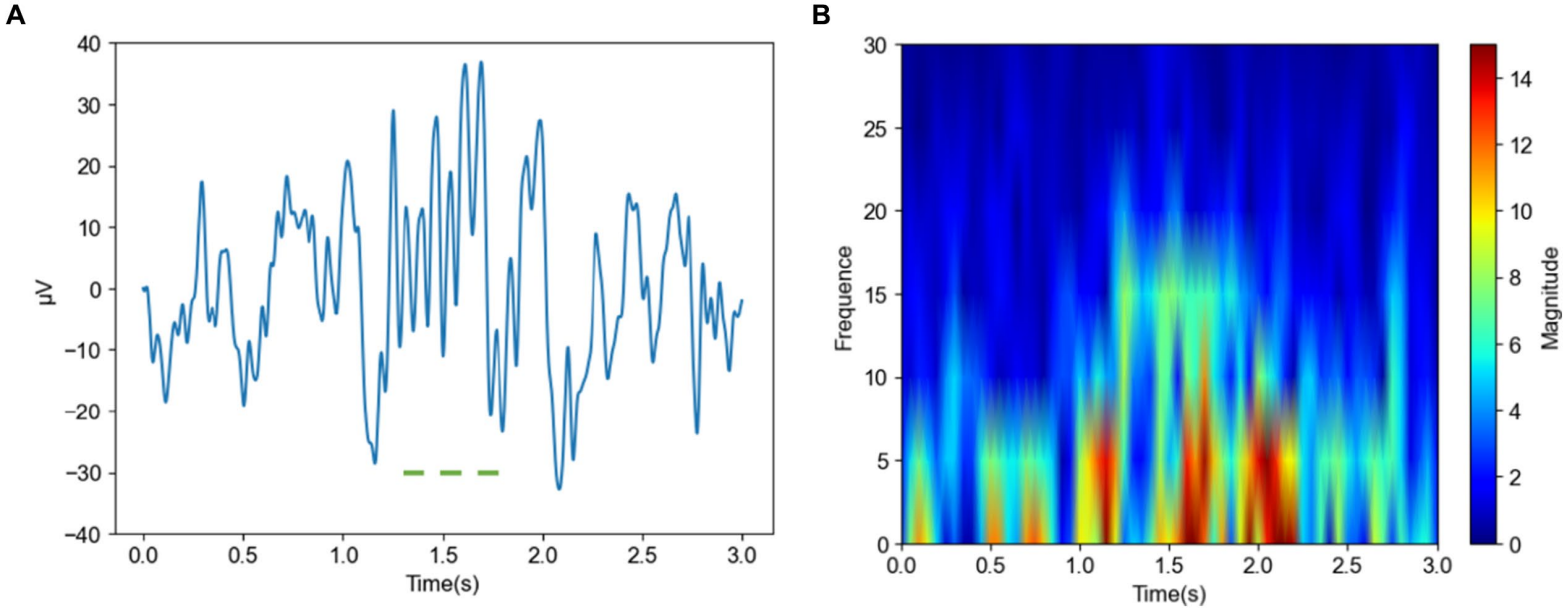
Time and frequency domain plots of a spindle in an insomnia subject **(A)** Time domain of a spindle **(B)** Frequency domain of a spindle.

Figure 4 shows images of a spindle in an insomniac subject. In the time domain image Figure 4A of the spindle in the insomnia patient, the waveform fluctuates greatly, from −10 to 40 μV (spanning 50 μV) and lasts for approximately 0.5 s. The sleep spindle of the insomnia subject is different in appearance, amplitude, frequency, and duration compared to those of the normal subjects. From the frequency domain plot for the insomniac participant shown in Figure 4B, compared with the sleep spindle of the normal subject plotted in Figure 3, the energy area is smaller, and the range of energy is smaller than that of the normal subject. However, the CNN can rapidly identify whether this is a sleep spindle based on the presence of the sigma band. Note that manual spindle detection in insomnia patients performed exclusively by experienced doctors or technicians would incur a huge workload.

## 3 Methods

### 3.1 Proposed model

**Figure 5:**
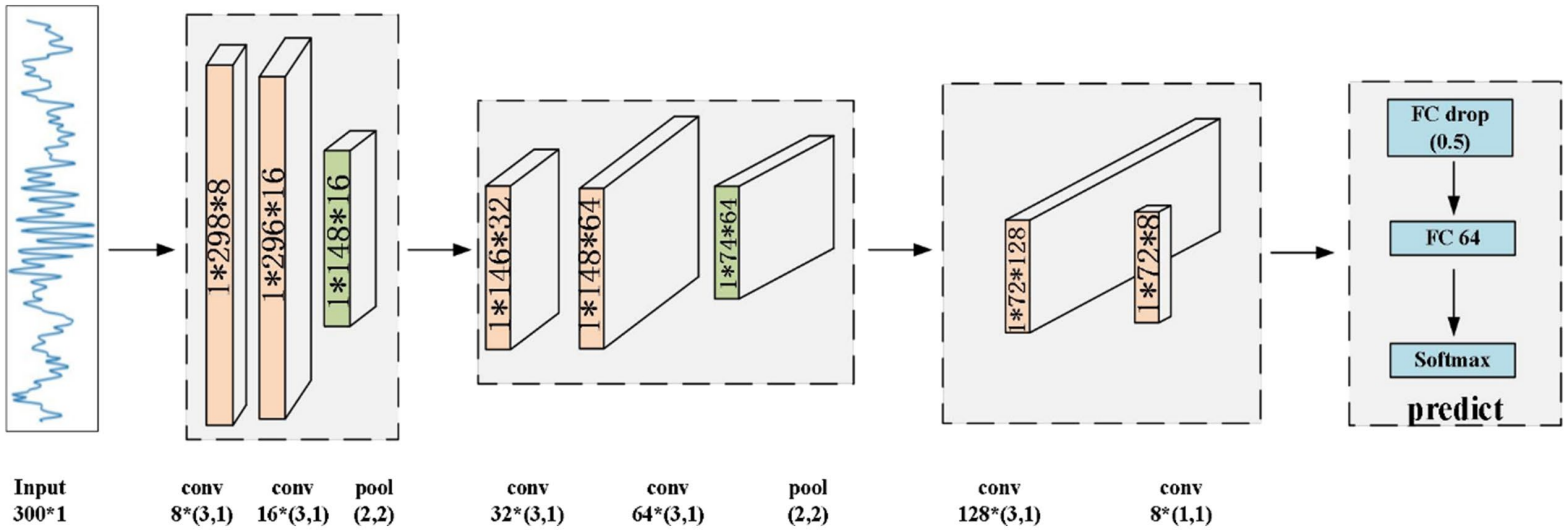
Architecture of the proposed CNN model.

In this section, we present the proposed CNN model to identify sleep spindles. The architecture of the proposed CNN model is shown in Figure 5. Here, the preprocessed data underwent processing by a CNN model for feature extraction and classification purposes. Initially, the EEG data passed through two convolutional layers for feature extraction, followed by an average pooling layer to diminish feature dimensions while preserving vital information. Then, the data were passed through two additional convolutional layers for deeper feature extraction, with the extracted features subsequently undergoing compression through an average pooling layer. Finally, the data were input to a convolutional layer for advanced feature extraction, and the resultant features were classified via fully-connected layers to obtain the final result. Through this hierarchical feature extraction and abstract representation, the proposed model could thoroughly explore the intrinsic patterns of input sleep spindles and perform classification.

The proposed CNN primarily comprises convolutional layers, rectified linear unit (ReLU) layers, batch normalization (BatchNorm) layers, average pooling layers, fully-connected layers, and a SoftMax layer. For the target EEG data, the convolution operation can effectively extract features from the signal. In the convolutional layers, the convolution operation relies on a sliding window, which allows the model to further extract the abstracted features.

The ReLU layer, is used to enhance the nonlinear expressive capability of a network. This function transforms negative input values to 0 while preserving positive input values, thereby enhancing the expressive capacity of the network. Compared to other activation functions, this unit provides better learning performance and faster convergence speed. The ReLU function is expressed as Eq. 1:


(1) $$\mathrm{ReLU}\left(x\right)=\max\left(x,0\right)$$

where x represents the input value.

The average pooling layer reduces the dimensionality of the EEG data, decreases its complexity, reduces the computational requirements, and enhances the model’s generalizability. The BatchNorm layer is a special normalization layer designed to address internal covariate shift and the gradient vanishing problem. It makes network training more stable and efficient by normalizing the data of each batch. Following feature extraction, the fully-connected layer is implemented to integrate these features and prepare them for final classification. Finally, the SoftMax layer maps the output of the fully-connected layer to a probability distribution, providing the final classification result.

### 3.2 Transfer learning in insomnia subjects

The core elements of transfer learning encompass the source and target domains. In this study, the data acquired from the healthy individuals served as the source domain and those acquired from the insomnia subjects served as the target domain. For the transfer learning experiment, we utilized 13,960 spindles and 13,960 nonspindles from the insomnia subjects. This experiment was performed to apply the knowledge learned from the CNN of normal individuals to realize the detection of spindles in the insomnia subjects.

In simple terms, transfer learning (Agarwal et al., 2021) refers to the use of methods that have already solved problems to address unresolved problems. This approach can significantly reduce time costs when solving problems while yielding relatively stable and reliable training results. In this experiment, transfer learning involved applying the features of spindles learned by the CNN in training to the data of the insomnia subjects. Spindles are considered an important factor in the study of sleep disorders in insomnia subjects. However, their extraction primarily relies on the experience of doctors, which is a time-consuming and laborious process. Using transfer learning, the detection results can be obtained rapidly, improving training efficiency and establishing a foundation for future clinical applications.

**Figure 6:**
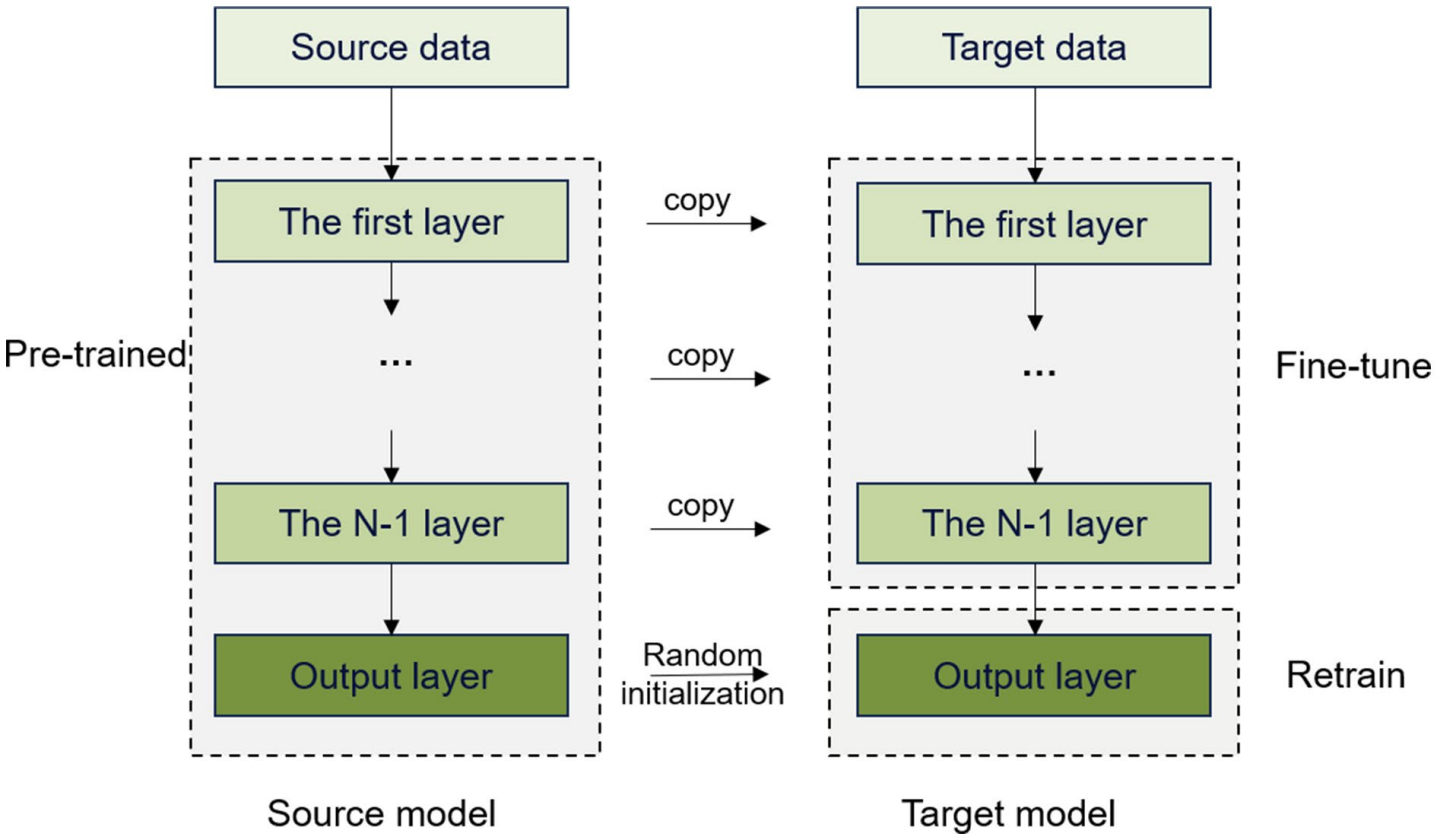
Transfer learning by fully transferring convolutional layers.

To assess the effectiveness of learning, we conducted two experiments to validate the effectiveness of using a pretrained model on the normal subjects. The first experiment involved transferring only the first four layers of the model and retraining and testing the remaining convolutional and classification layers. In the second experiment, the convolutional layers were transferred in full, with only the classification layer being trained and tested. Figure 6 shows the transfer learning method of fully transferring the convolutional layers of the model.

We trained the model using spindles acquired from the normal subjects, which were then transferred to the spindles of the insomnia subjects. This approach can avoid restarting the training process, thereby reducing both training time and computational costs. In addition, the model can utilize existing knowledge and experience to adapt to the new task quickly, which greatly improves learning efficiency and enhances generalizability to better adapt to different data distributions and scenarios.

### 3.3 Evaluation metrics

In this paper, the precision, recall, accuracy, F1 score, and the AUC are employed to evaluate the model. These metrics provide clear assessments of the performance of automated detection algorithms. And the equations are shown in Eqs. 2–5.

Accuracy is the ratio of the samples predicted correctly by the model to the total number of samples, showcasing the model’s overall predictive performance. Accuracy is calculated as follows.


(2) $$\mathrm{Accuracy}=\frac{TP+TN}{TP+FP+TN+FN}$$

Recall signifies the fraction of spindles detected correctly by the model, indicating its capability to identify all genuine instances.


(3) $$\mathrm{Recall}=\frac{TP}{TP+FN}$$

The F1 score is the harmonic mean of precision and recall, which is used to evaluate the performance of the model more comprehensively. If both precision and recall are high, the F1 score will also be high, indicating that the model’s performance is good.


(4) $$F1\;\mathrm{score}=2*\frac{\mathrm{Precision}*\mathrm{Recall}}{\mathrm{Precision}+\mathrm{Recall}}$$

Precision represents the proportion of samples judged by the model to be spindles that are indeed spindles.


(5) $$\mathrm{Precision}=\frac{TP}{TP+FP}$$

Here, true positive (TP) represents the count of positive samples correctly predicted as positive by the model, and true negative (TN) represents the count of negative samples correctly predicted as negative. False positive (FP) represents the count of negative samples predicted incorrectly as positive, and false negative (FN) indicates the count of positive samples predicted incorrectly as negative. The AUC, which stands for the area under the ROC (receiver operating characteristic) curve, assesses the model’s ability to discriminate between positive and negative samples. A model with an AUC closer to 1 indicates superior classification performance.

### 3.4 Experimental setup

To train and test the CNN, we set the number of epochs to 100 and the batch size to 128. We employed the AdamW optimizer with an initial learning rate of 0.1, which was adjusted to 0.75 times the original rate every three epochs. In addition, we conducted five-fold cross-validation. For a comprehensive validation of the model’s performance, the same five-fold cross-validation approach was employed during transfer learning using the same settings.

## 4 Results

### 4.1 Results of spindle classification in normal subjects

The proposed CNN model was used to classify spindles in the normal subjects. Here, five-fold cross-validation was conducted to validate model performance, and the results are given in Table 2.

**Table 2:** Five-fold cross-validation results for normal subjects.

	Accuracy (%)	Recall (%)	F1 score (%)	Precision (%)	AUC
1	**94.14**	94.70	94.17	**93.65**	**0.9843**
2	93.76	94.12	93.78	93.45	0.9817
3	93.15	94.07	93.21	92.36	0.9786
4	93.23	94.13	93.29	92.47	0.9803
5	94.10	**95.62**	**94.19**	92.80	0.9839
Average (standard deviation)	93.68 (0.42)	94.53 (0.59)	93.73 (0.42)	92.95 (0.52)	0.9818 (0.0022)

Bolded text indicates the best result in this column.

As shown in Table 2, the proposed classification model achieved an average accuracy of 93.68% with a standard deviation of 0.42% after five-fold cross-validation. The first fold exhibited the highest accuracy of 94.14%, which is 0.46% higher than the average. The lowest accuracy was observed in the third fold at 93.15%, which is 0.99% lower than that in the first fold and 0.53% below the average. The average recall from the five-fold cross-validation was 94.53% ± 0.59%, with the fifth fold achieving the highest recall at 95.62%, thereby exceeding the average by 1.09%. Conversely, the lowest recall was seen in the third fold at 94.07%, which is 1.55% lower than that in the first fold and 0.46% less than the average. The F1 score from the five-fold cross-validation was 93.73% ± 0.42%, with the fifth fold achieving the best performance at 94.19%. In addition, the average precision for spindle recognition in the five-fold cross-validation was 92.95% ± 0.52%, with the first fold achieving the best performance at 93.65% and the third fold obtaining the worst result at 92.36%. The average AUC was 0.9818 ± 0.0022.

By training and testing the proposed model, we achieved improved and stable results. The results demonstrated an improvement compared to those of similar previous CNN models and were relatively stable in the five-fold cross-validation (Table 2), with a small fluctuation in standard deviation. This validates the feasibility of the experimental results and verifies the usability of the experimental data.

Fine-tuning the parameters of the proposed CNN model, that improved the final results and realized high accuracy, providing a solid foundation for subsequent transfer learning tasks.

### 4.2 Classification results for insomnia subjects

The spindles of 10 insomnia subjects were classified by the proposed model, and the results of the five-fold cross-validation are shown in Table 3.

**Table 3:** Five-fold cross-validation results for insomnia subjects.

	Accuracy (%)	Recall (%)	F1 score (%)	Precision (%)	AUC
1	92.68	94.13	92.78	91.47	0.9789
2	92.87	94.48	92.99	91.53	0.9775
3	**93.57**	**94.73**	**93.64**	92.58	**0.9833**
4	93.36	94.09	93.40	**92.73**	0.9810
5	91.35	93.41	91.52	89.71	0.9749
Average (standard deviation)	92.77 (0.87)	94.17 (0.50)	92.87 (0.82)	91.61 (1.21)	0.9791 (0.0032)

Bolded text indicates the best result in this column.

As shown in Table 3, the average accuracy for the insomnia subjects reached 92.77%, with the best performance observed in the third fold at 93.57%. The worst accuracy was recorded in the fifth fold at 91.35%, which is 1.42% lower than the average. The average recall was 94.17% ± 0.50%, with the third fold performing the best at 94.73%, which is 0.56% higher than the average. The F1 score for the five-fold cross-validation was 92.87% ± 0.82%, with the third fold performing the best at 93.64%. The average precision of the model reached 91.61% ± 1.21%. In addition, the average AUC was 0.9791, with a standard deviation of 0.0032.

The results shown in Tables 2, 3 indicate that the classification results obtained for the normal subjects were generally better than those obtained for the insomnia subjects. This finding may be attributed to several factors. First, spindles from the normal subjects may possess more stable and consistent features, making them easier to distinguish, whereas spindles from the insomnia subjects may exhibit certain variations or abnormalities, making them more challenging to differentiate. In addition, the poor performance for the insomnia subjects could also be influenced by the relatively smaller number of spindles.

### 4.3 Classification results of transfer learning

The model trained on the data from the normal subjects was applied to the data of insomnia subjects and then fine-tuned. To determine the most suitable parameter transfer scheme to establish the spindle recognition model, we conducted different experiments focusing on the extent of model parameter transfer. These experiments included full transfer of the convolutional layers and transfer of only the first four convolutional layers. The results were then analyzed.

We transferred all convolutional layers, retrained the classification layer, and conducted five-fold cross-validation. The results are shown in Table 4.

**Table 4:** Results of transferring all convolutional layers.

	Accuracy (%)	Recall (%)	F1 score (%)	Precision (%)	AUC
1	91.48	**92.31**	91.56	90.82	**0.9717**
2	91.53	91.88	**91.57**	91.26	0.9703
3	91.00	91.78	91.08	90.39	0.9678
4	91.44	91.42	91.45	91.48	0.9702
5	**91.60**	91.10	**91.57**	**92.05**	0.9706
Average (standard deviation)	91.41 (0.24)	91.70 (0.46)	91.45 (0.21)	91.20 (0.64)	0.9701 (0.0014)

Bolded text indicates the best result in this column.

After transferring all convolutional layers, the average accuracy was 91.41% with a standard deviation of 0.24%. The best result was obtained in the fifth fold, reaching 91.60%, which is 0.19% higher than the average. The worst result was observed in the third fold at 91.00%, which is 0.60% lower than that in the fifth fold and 0.41% lower than the average. In terms of recall, the average value was 91.70% ± 0.46%. The highest recall value was obtained in the first fold at 92.31%, being 0.61% higher than the average. The lowest recall value was obtained in the fifth fold at 91.10%. In addition, the average F1 score for the five-fold cross-validation was 91.45% with a standard deviation of 0.21%. Note that the highest F1 score was observed in the second and fifth folds, reaching 91.57%, and the lowest F1 score was obtained in the third fold at 91.08%. The average precision was 91.20% ± 0.64%. The AUC obtained from the five-fold cross-validation reached an average of 0.9701 ± 0.0014. The highest AUC value was obtained in the first fold at 0.9717, being 0.0016 higher than the average, and the lowest AUC value was obtained in the third fold at 0.9678, which is 0.0039 lower than that in the first fold and 0.0023 lower than the average.

To further investigate the application of transfer learning in spindle detection, we transferred only the parameters of the first four layers and retrained the remaining convolutional layers and the classification layer. The results of the corresponding five-fold cross-validation are shown in Table 5.

**Table 5:** Five-fold cross-validation results for transferring only the first four layers.

	Accuracy (%)	Recall (%)	F1 score (%)	Precision (%)	AUC
1	**92.80**	**94.28**	**92.92**	91.59	**0.9782**
2	92.26	92.42	92.29	92.16	0.9741
3	91.62	93.46	91.78	90.17	0.9713
4	91.99	91.92	92.00	**92.08**	0.9742
5	92.21	92.81	92.27	91.73	0.9747
Average (standard deviation)	92.18 (0.43)	92.98 (0.92)	92.25 (0.43)	91.55 (0.81)	0.9745 (0.0024)

Bolded text indicates the best result in this column.

When transferring only the first four layers, the average accuracy was 92.18% with a standard deviation of 0.43%. The highest accuracy was achieved in the first fold at 92.80%, which is 0.62% higher than the average. The average recall was 92.98% ± 0.92%. The highest recall was obtained in the first fold at 94.28%, exceeding the average by 1.3%. In contrast, the lowest recall was observed in the fourth fold at 91.92%, which is 1.06% lower than the average. The average F1 score for the five-fold cross-validation was 92.25% with a standard deviation of 0.43%. The highest F1 score was observed in the first fold at 92.92%, and the lowest was observed in the third fold at 91.78%. The average precision was 91.55% ± 0.81%. In addition, the AUC obtained from the five-fold cross-validation had an average value of 0.9745 ± 0.0024. Here, the best result was achieved in the first fold, reaching 0.9782, which was 0.0037 higher than the average, and the lowest AUC was observed in the third fold at 0.9713, which was 0.0069 lower than that in the first fold and 0.0032 lower than the average.

Compared to fully transferring convolutional layers, the model with only the first four layers transferred demonstrated improvements in all evaluation metrics. This mode realized a 0.77% increase in accuracy, 1.28% increase in recall, 0.8% increase in F1 score, 0.35% increase in precision, and 0.0044 increase in AUC. These results suggest to some extent that the basic features extracted by the first four layers of the neural network are common to the spindles of the normal and insomnia subjects. Transferring only the first four layers may yield better results, and the latter layers of the neural network may focus more on extracting specific features unique to the spindles of the normal and insomnia subjects.

From the results given in Tables 2, 4, 5, it can be observed that whether conducting comprehensive transfer of convolutional layers or only transferring the first four convolutional layers, the overall values of the evaluation metrics for spindles of the insomniac subjects are reduced compared to those of the normal subjects. This is because the spindles of the normal subjects are relatively standardized and easy to distinguish in terms of amplitude and frequency. However, the spindles of the insomnia subjects may not be recognized due to significant changes in amplitude or failure to reach corresponding frequency indexes. Nevertheless, the results are still satisfactory.

From the results shown in Tables 3–5, it can be observed that the model trained and tested directly on the data from the insomniac subjects yielded slightly better performance compared to using transfer learning because there are certain differences in the feature distributions of the data from the insomnia and normal subjects. Transfer learning can partially compensate for the differences between different datasets; however, it cannot completely eliminate such differences. In addition, the data from the insomnia subjects may contain some features that are specific to insomnia, and the model trained directly on this dataset may better capture these features, achieving slightly better performance in the classification of the insomnia subjects.

From the results shown in Tables 4, 5, it can be observed that the results obtained by transferring only the first four convolutional layers are better than those obtained by transferring all convolutional layers. This may be due to the fact that the first four convolutional layers extract the basic features of the spindles, which are consistent across the spindle data from the normal and insomnia subjects. As the number of layers increases, higher-level features become more specific and may be more closely related to the subjects’ state (either normal or insomnia subjects). The neural network may be able to extract unique features of the spindles from the normal and insomniac subjects; thus, full transfer may not perform effectively.

## 5 Discussion

In this study, we collected EEG data from 30 subjects, including 20 normal and 10 insomniac subjects, with spindles annotated for the C3 and C4 channels of all subjects. Separate analyses were conducted on the spindles of normal and insomniac subjects. In addition, a CNN model is proposed, and cross-subject training and testing were conducted using data from the normal subjects. The results of five-fold cross-validation demonstrated an accuracy of 93.68%, average recall of 94.53%, F1 score of 93.73%, average precision of 92.95%, and AUC of 0.9818. Similarly, cross-subject training and testing were conducted using spindles from the insomnia subjects; the results of five-fold cross-validation demonstrated that the accuracy of the model reached 92.77%, with an average recall of 94.17%, F1 score of 92.87%, precision of 91.61%, and AUC of 0.9791. These results demonstrate the effectiveness of the data collected in this study and the proposed CNN model.

We also investigated the application of transfer learning in spindle analysis using a CNN model trained on data from the normal subjects to detect spindles in insomnia patients. When transferring all convolutional layers, the model achieved an average accuracy of 91.41%, an average recall of 91.70%, an average F1 score of 91.45%, and an average precision of 91.20%. In addition, the AUC of the model was 0.9701. These results indicate that transfer learning can be applied to spindle detection in both normal and insomnia subjects and can obtain satisfactory results. To further investigate the impact of different transfer degrees on the results, an experiment was conducted by only transferring the first four layers. Here, we found that the average accuracy in five-fold cross-validation was 92.18%, the recall was 92.98%, the average F1 score was 92.25%, the precision was 91.55%, and the AUC was 0.9745. The transfer learning results indicate that transferring only the first four layers yields better results compared to transferring all of the convolutional layers. This may be because the features learned by the initial layers are more general and encompass common features present in spindles, while the subsequent convolutional layers may be more specialized to learn specific features that are relevant to the original task. Spindle data from normal and insomnia subjects may be similar in some common features; thus, transferring the initial layers selectively can utilize the generalized features of the pretrained model effectively, thereby enhancing the model’s performance on the data from insomnia subjects.

The proposed method has exhibited favorable outcomes in spindle identification for individuals with and without sleep disorders, as well as in the domain of transfer learning. Compared to the SpindleNet model (Kulkarni et al., 2019), the F1 score obtained by the proposed model exhibits an improvement of approximately 17%. In addition, in contrast to an existing fusion algorithm (Chen et al., 2023), the accuracy of the proposed model demonstrates an enhancement of approximately 2%. These findings signify that the proposed model exhibits superior performance and efficacy in the spindle recognition task. Using transfer learning, the knowledge obtained from a model trained on spindles from normal subjects can reduce the time and computational resources required to train a model for a new task significantly. By fine-tuning the model using spindles from insomniac subjects, the model can better adapt to the specific task, thereby enhancing the model’s generalizability on unknown data.

There are some limitations to the proposed methods. Firstly, this study only collected data from 30 participants and extracted spindles for analysis and classification. Thus, the results may be influenced by the small sample size. Secondly, this study only focused on analyzing spindles from the C3 and C4 channels. Therefore, in the future, it will be necessary to expand the sample size to improve the reliability and generalizability of the results obtained in the current study. In addition, we will consider additional channels to further analyze the differences in spindle characteristics among different subjects. Furthermore, the subsequent work could incorporate additional sleep spindle features, e.g., amplitude, density, and frequency, to explore their role and significance in the sleep process.

The proposed CNN model performed well in the spindle wave recognition task, achieving excellent results for normal participants and insomnia participants, and demonstrating potential in transfer learning. The findings of this study provide strong support and reference for future research and the practical application of similar spindle recognition technologies.

## 6 Conclusion

In this paper, we propose a CNN model to classify sleep spindles in healthy individuals, yielding an accuracy of 93.68%, a recall of 94.53%, and an AUC of 0.9373. In addition, the proposed model achieved a classification accuracy of 92.77% on insomnia subjects. These outcomes underscore the efficacy of both the collected dataset and the proposed CNN model.

Using transfer learning, the CNN model trained on spindle data from healthy subjects was transferred to spindle data from individuals with insomnia. This approach facilitated a faster training process, conservation of both computational resources and time, and bolstered the model’s generalizability, thereby enhancing performance on new tasks. When transferring all convolutional layers, the model obtained an average accuracy of 91.41%, while transferring only the initial four convolutional layers resulted in an average accuracy of 92.18%. These results highlight the applicability of transfer learning in spindle recognition, with superior recognition performance observed when transferring only a subset of the convolutional layers. These findings exhibit potential for advancing the automated detection of clinical sleep spindles, minimizing labor costs, aiding in sleep disorder diagnoses, and elevating diagnosis and treatment standards.

## Data availability statement

The data analyzed in this study is subject to the following licenses/restrictions: data archiving is not mandated but data will be made available on reasonable request. Requests to access these datasets should be directed to Evanliangjun@tmu.edu.cn.

## Ethics statement

The studies involving human participants were reviewed and approved by Xuanwu Hospital, Capital Medical University. The studies were conducted in accordance with the local legislation and institutional requirements. The participants provided their written informed consent to participate in this study.

## Author contributions

JL: Writing – original draft. AB: Writing – original draft. YS: Writing – original draft. JW: Writing – original draft. ZA: Writing – original draft. XW: Writing – original draft. JG: Writing – original draft. LF: Writing – original draft. CW: Writing – original draft. BJ: Writing – original draft. ZW: Writing – original draft.
